# Heterogeneous Clustering of Multiomics Data for Breast Cancer Subgroup Classification and Detection

**DOI:** 10.3390/ijms26041707

**Published:** 2025-02-17

**Authors:** Joseph Pateras, Musaddiq Lodi, Pratip Rana, Preetam Ghosh

**Affiliations:** 1 Department of Computer Science, Virginia Commonwealth University, Richmond, VA 23284, USA; pghosh@vcu.edu; 2 Integrative Life Sciences, Virginia Commonwealth University, Richmond, VA 23284, USA; lodimk2@vcu.edu; 3 Department of Computer Science, Old Dominion University, Norfolk, VA 23529, USA

**Keywords:** multiomics data integration, gene expression profiling, cancer subtyping, survival analysis, machine learning methods

## Abstract

The rapid growth of diverse -omics datasets has made multiomics data integration crucial in cancer research. This study adapts the expectation–maximization routine for the joint latent variable modeling of multiomics patient profiles. By combining this approach with traditional biological feature selection methods, this study optimizes latent distribution, enabling efficient patient clustering from well-studied cancer types with reduced computational expense. The proposed optimization subroutines enhance survival analysis and improve runtime performance. This article presents a framework for distinguishing cancer subtypes and identifying potential biomarkers for breast cancer. Key insights into individual subtype expression and function were obtained through differentially expressed gene analysis and pathway enrichment for BRCA patients. The analysis compared 302 tumor samples to 113 normal samples across 60,660 genes. The highly upregulated gene COL10A1, promoting breast cancer progression and poor prognosis, and the consistently downregulated gene CDG300LG, linked to brain metastatic cancer, were identified. Pathway enrichment analysis revealed similarities in cellular matrix organization pathways across subtypes, with notable differences in functions like cell proliferation regulation and endocytosis by host cells. GO Semantic Similarity analysis quantified gene relationships in each subtype, identifying potential biomarkers like MATN2, similar to COL10A1. These insights suggest deeper relationships within clusters and highlight personalized treatment potential based on subtypes.

## 1. Introduction

In medical data sciences, multimodal data integration is an important tool for researchers to gather important conclusions from otherwise intractable sources of information. Diverse sources of biological data require novel methods for information reconciliation. For insights into genetic diseases, sequencing technology has created a treasure trove of minable data. Genome, transcriptome, epigenome, and more types of -omics data can be sequenced to the daily tune of thousands of specimen profiles by a single machine. Genomics is defined as the study of organisms’ whole genomes; within genomics, variants and features are analyzed at the organism and population levels in order to identify genes that exhibit certain traits. Similarly, transcriptomics allows researchers to understand the presence or absence of a certain gene within a genome, which leads to analysis such as differential expression. Epigenomics is the study of macromolecules that may affect the silencing or expression of certain genes [1,2]. Employing these data has created truly novel avenues toward disease insights, subclassification, and biomarker prediction. Combining patient -omics profiles with clinical data allows learning machines to intuit hidden relationships across data modalities.

Perhaps more so than other human disorders, cancer is an essential target for multiomics data integration. Leveraging omics data is crucial to understanding the biological mechanisms of cancer. Genomic data such as whole-genome sequencing (WGS) have been used to identify cancerous genome variants across populations, leveraging the information from genome-wide association studies (GWASs) [3]. Additionally, RNA sequencing technology has helped uncover genes that are differentially expressed in cancer patients, leading to drug targets and other therapeutic information [3]. At an epigenome level, DNA methylation data have been used to accurately predict the risk of neoplastic recurrence. Methylation biomarker expression was analyzed across several patients, and this information was leveraged to make predictions according to a patient’s potential cancer susceptibility [4]. To name only a few important data collectors, the Clinical Proteomic Tumor Analysis Consortium (CPTAC), the International Cancer Genomics Consortium (ICGC) (CPTAC, https://proteomics.cancer.gov/programs/cptac (accessed on 1 November 2023)), and the Cancer Cell Line Encyclopedia (CCLE) (ICGC, https://cancergenomics.org (accessed on 1 November 2023)) provide data for dozens of primary cancer types (CCLE, https://sites.broadinstitute.org/ccle (accessed on 1 November 2023)) across tens of thousands of samples. The data for this study were collected from the largest repository of multiomics cancer data, The Cancer Genome Atlas Program (TCGA). Since 2006, TCGA has obtained over 20,000 samples across 33 cancer types. This publicly available dataset boasts over 2.5 petabytes of genomic, epigenomic, transcriptomic, and proteomic data. Consisting of cross-cancer analyses and littered with novel tumor classifications, TCGA published their “Pan-Cancer Atlas” flagship paper employing the entire TCGA database [5]. These data have become a valuable tool for disease classification, subtyping, and biomarker prediction used to gain insights into disease biology. As such, tools for data interpretation, analysis, and the visualization of multiomics data have exploded. Consequently, the wide variety of multiomics data integration approaches allows novel mathematical formulations to influence existing techniques.

-Omics data may be present in several forms or gathered by various methodologies. A multiomics approach combines information across modalities for a more informed analysis. For disease subclassification, there are many generic ways to utilize these multimodal data. Methods vary in how and when data are combined across -omics, and they feature various downstream optimization techniques. Joint or shared variable models combine -omics data, usually supplemented with feature selection, before the learning process. iCluster and its many variants [6,7] represent a popular method for joint -omics optimization with shared factors. Optimization is performed on latent variables shared among all data types. While iCluster utilizes an expectation–maximization routine for subtype optimization, various techniques can be utilized. Another method, intNMF [8], employs integrative methods for shared factors. However, for optimization, intNMF uses non-negative alternating least squares for block coordinate descent. Current joint variable methods are limited by the fidelity of their feature selection and the computational cost of handling large, merged sets. -Omics-specific factors might first learn clusters on individual data types before performing consensus clustering. The MOFA (Multi-Omics Factor Analysis) [9] method for individual factor multiomics data integration is designed to capture shared latent factors that explain the variation across multiple data types, allowing the analysis of associations of learned factors within the data. Often, individual factors are limited by the knowledge consolidation process and are limited by the loss of information among shared factors. As a recent effect, novel network representations of genomic data have enabled graph-based learning. Graph convolutional neural networks are a powerful method for multiomics data integration that leverages the underlying network structure between biological entities (e.g., genes, proteins) to capture intricate relationships across different -omics, like MOGONET [10]. By treating multiomics data as a graph, this approach can effectively learn and combine information from various sources, thus enabling comprehensive analysis and revealing hidden biological interactions within complex datasets.

Crucially, multiomics data integration must confront the difficulty of extreme dimensionality. Containing thousands of features, many such features are considered redundant, noisy, or otherwise irrelevant [11,12,13]. Therefore, reducing the dimensionality of genomic data effectively is a major concern. Typical methods for dimension reduction include Canonical Correlation Analysis (CCA) [14,15], Partial Least Squares (PLS) [16,17], and other variations of linear projection models. Unsupervised statistical methods like Principal Component Analysis (PCA) [18,19] are also adopted for -omics problems. Dimension reduction techniques are often chosen for compatibility with a specific downstream analysis, like disease subtyping. Techniques like Non-negative Matrix Factorization (NMF) [20,21] inherently cluster data during the reduction. Further, nonlinear methods for feature selection, including DTA [22], employ a *k*-cover scheme for maximizing patient similarity in selecting features for disease and subtyping analyses. In recent efforts, deep encoder/decoder architectures have been employed for the reduction in the genomic feature space. Franco et al. [23] provide a comparative study of deep autoencoders for multiomics data integration.

To achieve the goal of multiomics data integration, knowledge from disparate feature sets must be combined. This problem’s several -omics are modeled *jointly*. This is achieved by combining the multimodal data before—or contemporaneously with—learning via clustering. Alternative and related methods abound. TCGA authors [24] developed a probabilistic graph-based model employing directed feature graphs. This study used copy number variation (CNV) and gene expression -omics for glioblastoma multiforme. Using copy number and gene expression modalities, Chin et al. [25] and Aguirre et al. [26] applied data fusion via Bayesian nonparametric Dirichlet modeling to determine amplifications and deletions in breast cancer and pancreatic adenocarcinoma, respectively. An important distinction for joint latent variable models in multiomics data integration is the ability to learn from each -omics type with shared or mixed factors, instead of learning separately and later reconciling knowledge. Shen et al. propose iCluster, a joint latent variable model that employs expectation–maximization for latent distribution optimization [6] in performing subtyping for breast and lung cancers and glioblastoma. Further, iClusterPlus [27], which employs generalized linear regression for the formulation of a joint model, incorporates the lasso [28] regularization routine into the expectation–maximization process for increased performance. Subramanian et al. [29] provide an extensive review of multiomics data integration techniques.

This work aims to showcase a joint latent variable model for disease subtyping using multiomics patient profiles, employing a novel expectation–maximization routine. This improved routine, borrowed from other implementations of latent variable modeling, improves on previous methods for latent -omics variable modeling in terms of runtime and survival analysis. This work also identifies potential biomarkers of invasive breast carcinoma, leading to functional insights into each subtype established from this novel method. Further, the same method employed for subtyping in breast cancers is applied to glioblastoma multiforme survival analyses.

## 2. Results

**Figure 1:**
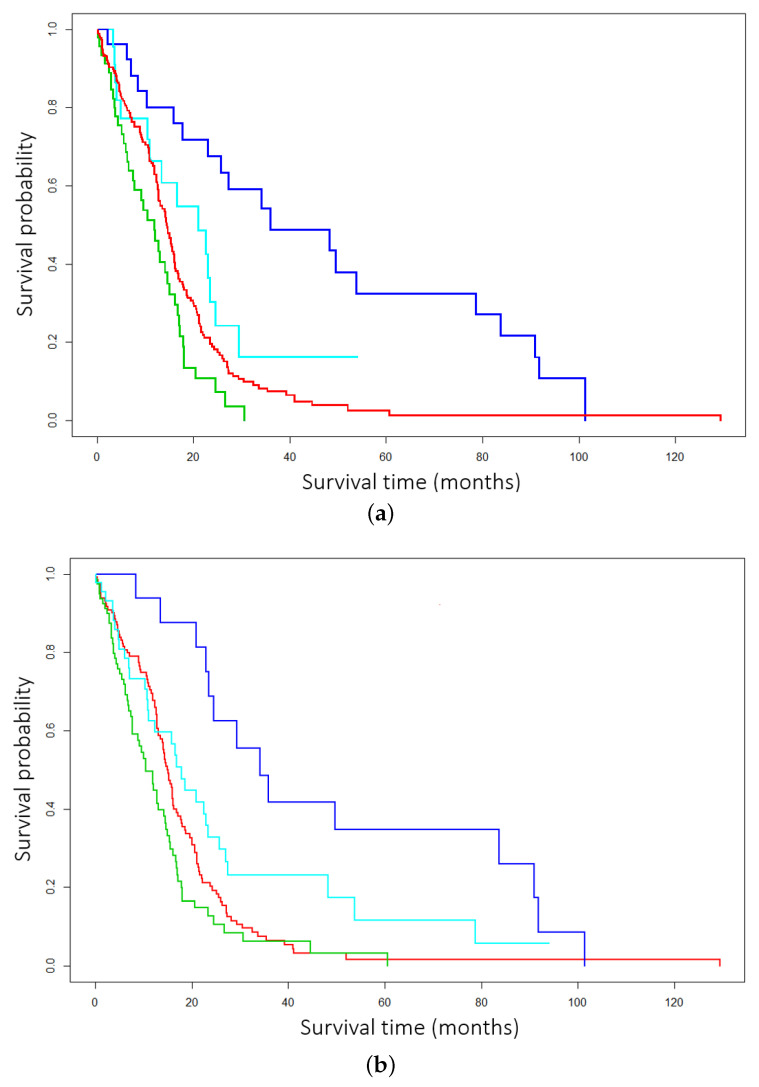
Comparison across expectation–maximization (EM) routines in the data-centric expectation–maximization (DCEM) package. (**a**) GBM subtyping with DCEM employing the improved data-centric EM* routine. (**b**) GBM subtyping with DCEM employing the traditional EM routine.

First, utilizing both EM and EM*, DCEM was employed to cluster multiomics data from The Cancer Genome Atlas glioblastoma (GBM) data repository. Both EM and EM* underwent identical preprocessing. First, feature selection was performed via Cox by selecting the most relevant features. Next, both methods were trained with multiomics data by stacking DNA, mRNA, and miRNA -omics. In Figure 1a,b the EM* routine achieves a *p*-value of 0.000000211 from the log-rank test, and the traditional EM scores a *p*-value of 0.0000344 from the log-rank test—in this instance of DCEM clustering, with three types of data and 276 patients. Here, Kaplan–Meier curves are an important piece of evidence by which the efficacy of the EM* algorithm is determined. For both BRCA and GBM, four clusters were used for the primary study. Four clusters enable comparison to cutting-edge techniques and popular benchmarking subtypes. Additionally, particularly distinct subgroups—usually distinguished by low survivability—can be verified. The four clsuters on the graph are arbitrarily assigned colors to denote to survival probabilty across groups.

Next, while performing subtyping on the TCGA-GBM dataset, the robustness of EM* to incomplete patient data was examined. *p*-values from the log-rank test as a function of randomly removed patients are displayed in Table 1. The average *p*-values over five different trials are provided in Table 1. For each trial, a random selection of patients is withheld from the training set. Patients are removed by selecting their indices employing the R runif() function, which selects patient indices from the uniform distribution on the simplex [30]. While the deviation among survival analysis trials increases as patients are removed, successful subtyping for survival analysis is still possible with substantial patient loss.

**Figure 2:**
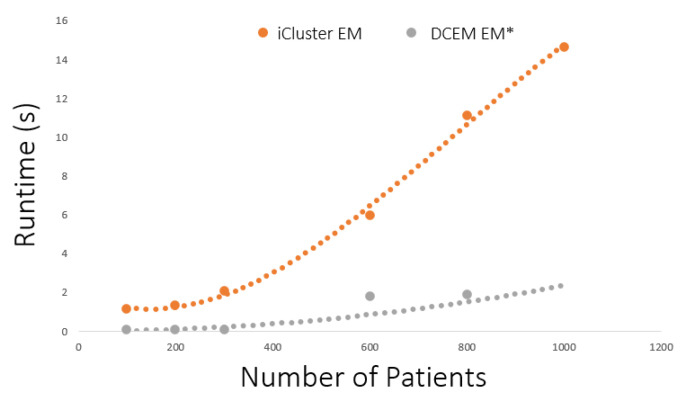
Runtimes in seconds of the expectation–maximization (EM) routine in iCluster and the EM* routine in the data-centric expectation–maximization package. Data points indicate a recorded run of the expectation–maximization routine in each method. Dotted lines indicate the best fit for runtime observations.

Finally, a major contribution of the novel EM* algorithm for multiomics data integration is the ability to alleviate computational burden. In Figure 2, the runtime expense associated with the EM* algorithm and the standard EM process employed by other multiomics tools like iCluster is explored. The runtime improvement associated with the data-centric EM* routine is shown by the disparity in cost incurred by introducing an increasing number of factors. The burden of large feature sets for EM* is less than for the traditional EM routine in iCluster because the data-centric approach iteratively excludes less expressive factors. While it is shown that the best subtyping for survival analysis or disease insights must include relevant feature selection, Figure 2 shows potential scalability to large feature sets with a relatively inexpensive computational burden.

**Figure 3:**
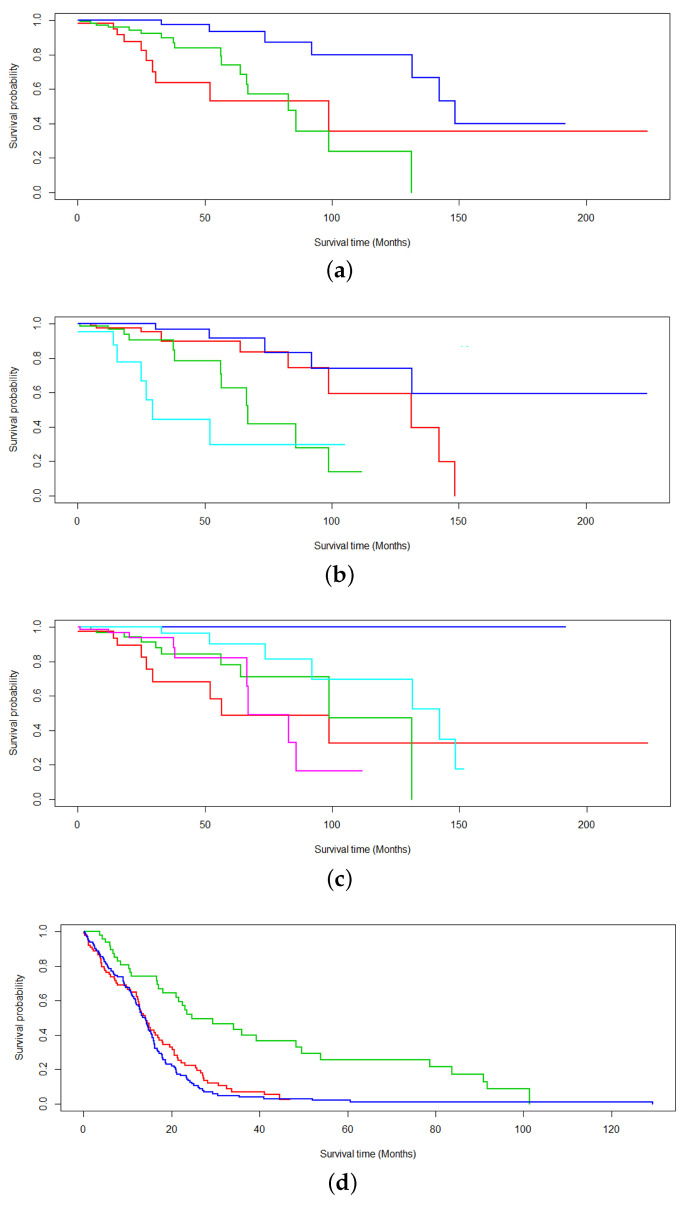

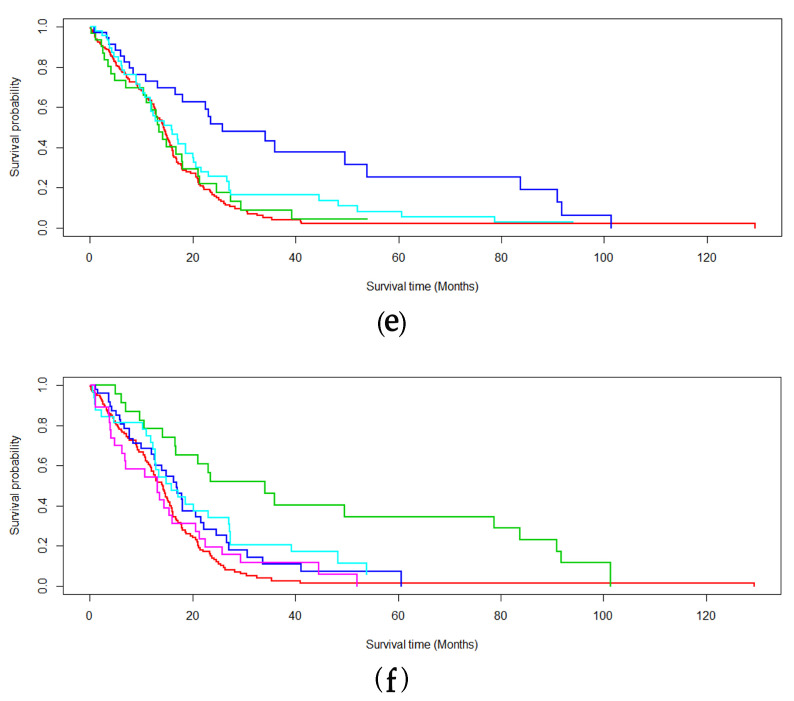
Survival analysis probing the robustness of the data-centric expectation–maximization (DCEM) algorithm to variable *k* subtyping. Values of k=3,4,5 are proposed for the number of subtyped (**a**–**c**) BRCA -omics and (**d**–**f**) GBM data.

Additionally, Figure 3 studies the robustness of EM* for multiomics survival analysis as values of *k* are toggled. Here, *k* denotes the number of latent variables chosen to model the subtypes in the expectation–maximization scheme. Values of k=3,4,5 are studied for TCGA-BRCA and TCGA-GBM. The respective average *p*-values recorded for each survival analysis are 0.00042 (Figure 3a), 0.0000416 (Figure 3b), 0.0152 (Figure 3c), 0.0000004 (Figure 3d), 0.000523 (Figure 3e), and 0.000121 (Figure 3f).

**Figure 4:**
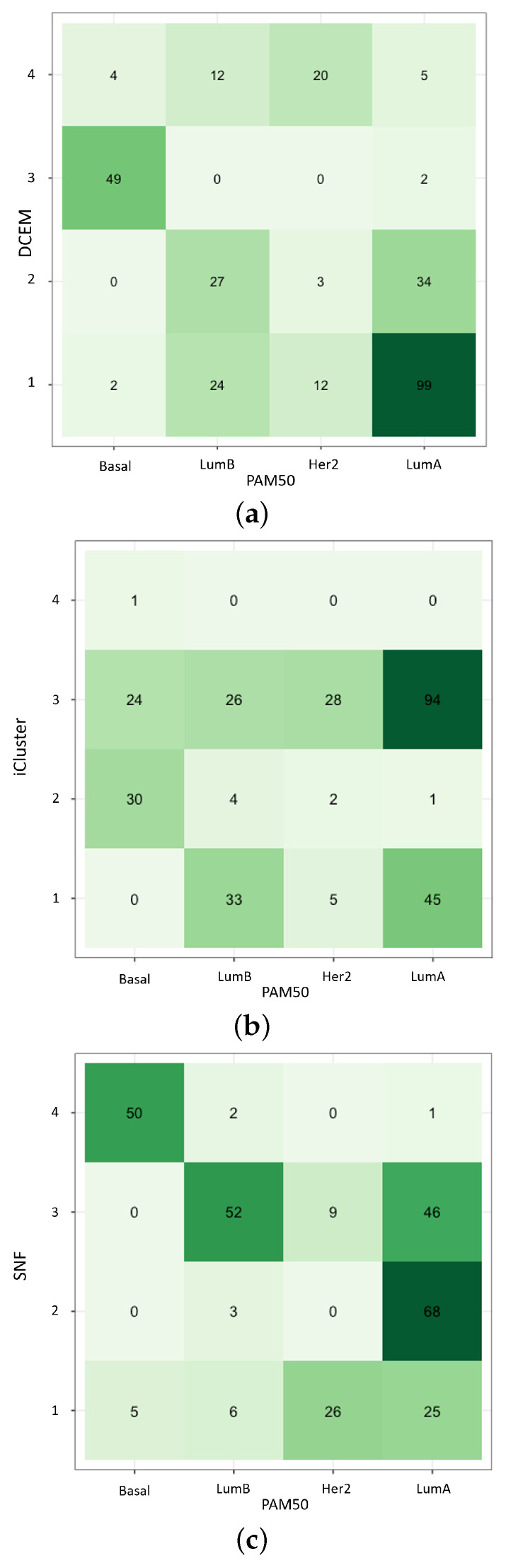
Concordance with PAM50 subtypes for (**a**) data-centric expectation–maximization (DCEM) routine, (**b**) iCluster, and (**c**) Similarity Network Fusion (SNF). Here, the crossover between several benchmark multiomics data integration methods, the EM* method, and the established PAM50 subtypes is quantified. Here the darker hues correspond to an increase in magnitude of the labeled data.

Finally, while using DCEM with EM* on the TCGA-BRCA multiomics dataset, the concordance of various methods is compared to the PAM50 benchmarking subtypes: LumA, LumB, Her2, and Basal. The Pam50 subtypes are available for download using the TCGAbiolinks package [31]. Other multiomics frameworks utilized include the Cancer Subtypes package built-in ExecuteiCluster [6] and ExecuteSNF [32] functions on DNA and mRNA -omics. Most notably, in Figure 4, concordance with PAM50 varies among methods—each correlating closely in different ways—but each achieves strong survival probabilities. Most notably, DCEM performs equally well at subtyping the particularly separable BRCA subgroup: Basal. Additionally, DCEM improves upon the difficult task of distinguishing the LumA from the LumB subtypes.

Key biological insights regarding individual subtype expression and function were obtained through differentially expressed gene analysis and pathway enrichment. This was undertaken for BRCA patients. There was not a sufficient amount of data to complete this analysis for GBM patients. To conduct the differentially expressed gene analysis for BRCA, 302 tumor samples were compared to 113 normal samples across 60,660  genes.

The pathway enrichment identified clear differences in the functionalities of each subtype. While the differentially expressed genes with the highest log fold change at either extreme tended to be similar across subtypes, inconsistencies in other differentially expressed genes seemingly changed the overall function of each subtype.

**Figure 5:**
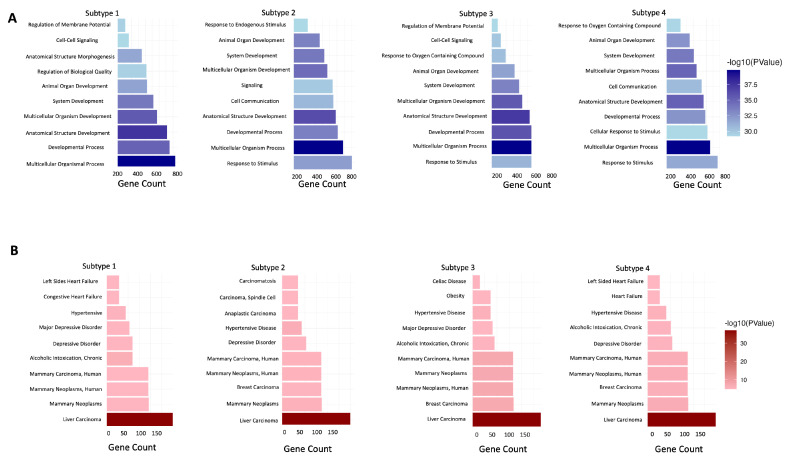
GOBP and DISGINET pathways for each of the four BRCA subtypes from the EM* method. (**A**) GOBP pathways for each BRCA subtype. (**B**) DISGINET pathways for each BRCA subtype.

The pathway enrichment for each subtype from BRCA tumor patient data can be seen in Figure 5. The four groups informing the subtype analyses are the DCEM with EM* routine found in Figure 3b. Across subtypes, there is an overrepresentation of developmental pathways. This similarity is an expected result, as the differentially expressed gene profiles across subtypes were similar as well. However, there are some notable differences in the overexpressed pathways between subtypes. While there are developmental pathways consistent across the four subtypes, there are some subtle differences. Subtypes 1 and 3 showed overrepresentation for the Regulation of Membrane Potential and Cell–Cell Signaling pathways. Subtype 2 demonstrated overrepresentation for the Response to Endogenous Stimulus pathway. The overlapping pathways between some subtypes, as well as some unique pathways, show that some subtypes are more functionally related than others, based on their specific differentially expressed genes.

Many patients diagnosed with BRCA and its corresponding subtypes are predisposed to several other cancer types as well. Specifically, many patients have demonstrated a higher risk of liver carcinoma after being diagnosed with breast cancer [33]. Strikingly, 40–50% of women diagnosed with breast cancer will have liver metastasis [34]. This may be due to the fact that common biomarkers for breast cancer, such as BRCA1 and BRCA2, have been linked to liver carcinoma as well. Therefore, the two cancer types are highly related and share many similar features [35]. It is reasonable to expect that BRCA biomarkers, as identified by this study, will also be similar to liver carcinoma biomarkers and, subsequently, their pathways.

Additionally, we performed disease-specific pathway enrichment using the DISGINET pathway database in DAVID [36]. The purpose of this analysis was to determine each subtype’s relationship to breast cancer. We note that each subtype showed enrichment for Mammary Carcinoma, Mammary Neoplasms, and Breast Carcinoma. This shows that, despite have unique DE gene profiles, as showcased in the GOSemSim analysis, they are still associated with breast cancer. This serves as an additional verification step for the biological relevance of the subtypes.

**Figure 6:**
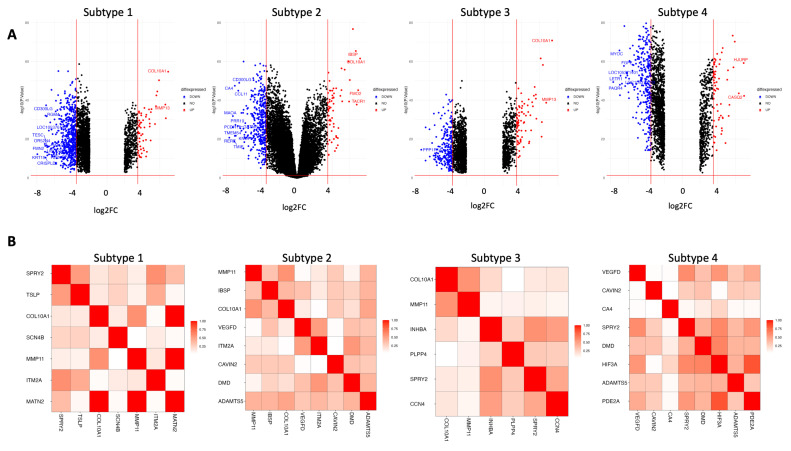
Volcano and GOSemSim plots for each BRCA subtype. (**A**) Volcano plots for each BRCA subtype. (**B**) GOSemSim plots for each BRCA subtype.

Figure 6 depicts the log fold change against the negative log base 10 adjusted *p*-value for each of the four subtypes for BRCA in the form of a volcano plot. The highly upregulated genes tend to be similar, with COL10A1 being significantly upregulated in three out of four subtypes. Additionally, CDG300LG featured consistently as a downregulated gene in three out of four subtypes.

The relationship between genes in each subtype was quantified through GO Semantic Similarity analysis [37]. The GO Semantic Similarity matrix for each BRCA subtype is depicted in Figure 6. The key takeaway from this analysis is the genes with high semantic similarity, which may reveal potential biomarkers and regulators for BRCA that are not currently well established in the existing literature. One way of achieving this is to identify genes with a high functional similarity with already existing BRCA biomarkers. Insights on a subtype level from differentially expressed gene and pathway enrichment analyses help to identify relationships between subtypes and can help inform treatment options for specific patients by subtype.

## 3. Materials and Methods

### 3.1. Data

The data for this study were collected from the largest repository of multiomics cancer data, The Cancer Genome Atlas Program (TCGA). Since 2006, TCGA has obtained over 20,000 samples across 33 cancer types. This publicly available dataset boasts over 2.5 petabytes of genomic, epigenomic, transcriptomic, and proteomic data. Consisting of cross-cancer analyses and littered with novel tumor classifications, TCGA published their “Pan-Cancer Atlas” flagship paper employing the entire TCGA database [5]. The -omics data employed in our study are of three types, DNA methylation, gene expression quantification, and miRNA gene quantification, for two different cancer types: glioblastoma multiforme (GBM) and invasive breast carcinoma (BRCA). DNA methylation data were processed with the Illumina human methylation 27 platform or the Illumina human methylation 450 platform (Illumina, San Diego, CA, USA). GBM was analyzed by following the SeSAMe methylation beta estimation workflow. Gene expression quantification was based on transcriptome profiling performed with the RNA-Seq experimental strategy. mRNA quantification followed the STAR-Counts workflow. miRNA gene expression quantification was based on transcriptome profiling performed with the miRNA-Seq experimental strategy and followed the BCGSC miRNA profiling workflow. For BRCA, 302 patients with matching data were obtained, and for GBM, 276 were obtained. For each patient, clinical information was also obtained from TCGA.

### 3.2. Feature Selection

**Figure 7:**
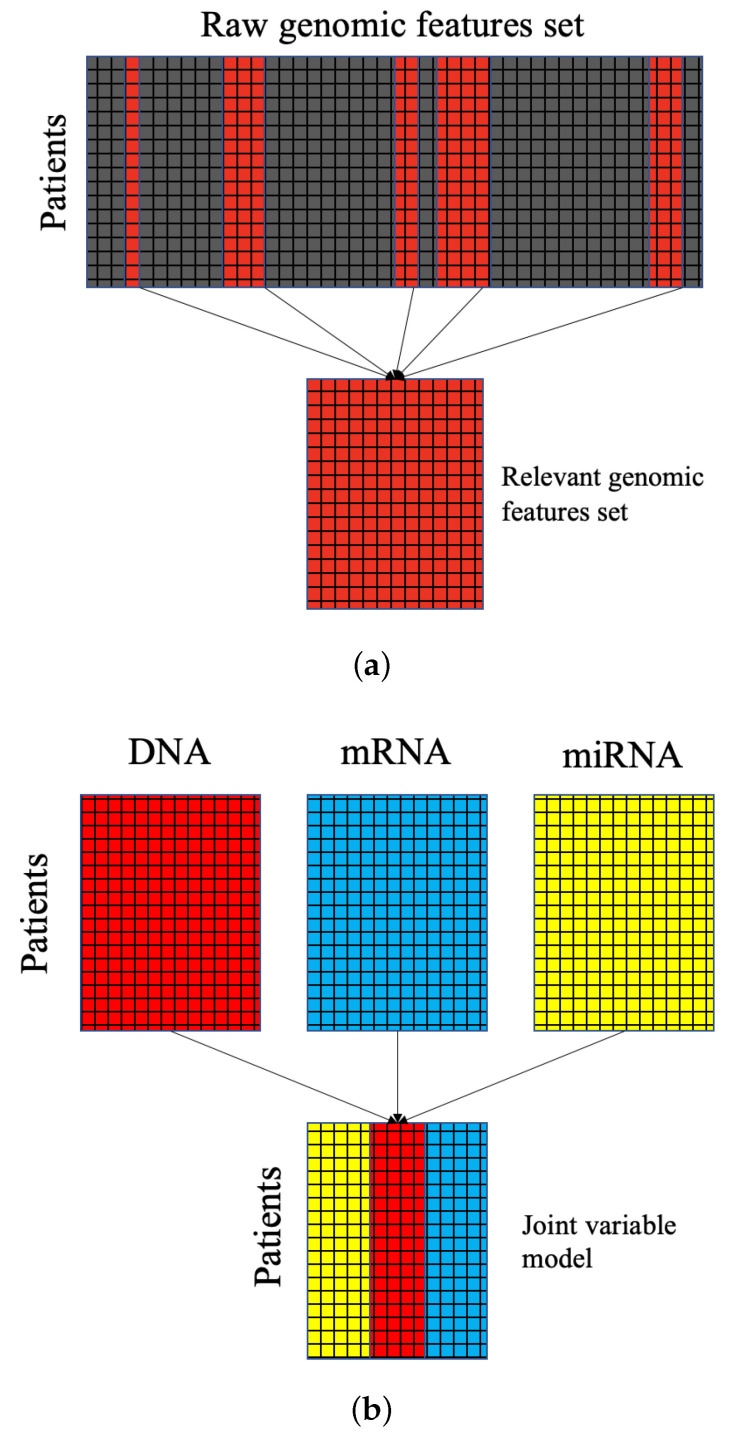
(**a**) A diagram of the -omics dimensionality reduction process via feature selection. Relevant features are highlighted and selected, while redundant and noisy features are removed. (**b**) A diagram of -omics stacking for joint latent variable modeling where features belonging to each down-selected -omics set are modeled together.

This study aims to learn from at least 3-omics of data: DNA methylation, mRNA, and miRNA. Each set alone contains a feature space that is too large for traditional approaches to cluster reliably. Thus, feature selection must be performed. To this end, we employed feature selection methods found in the *CancerSubtypes* [38] package. *CancerSubtypes* enables several feature selection methods: feature selection based on variance, based on median absolute deviation, and using Principal Component Analysis. The most efficacious of these methods employed in our study was *FSbyCox*—feature selection based on a Cox regression model. The Cox model, or the proportional hazard model, is a statistical approach to survival analysis. Based upon proportional survival assumptions, features are selected based on their relation to survival results for each sample. Therefore, features selected for clustering contain important biological relevance. Figure 7a gives an example schematic of the feature selection process—which is performed for each -omics data type.

### 3.3. Joint Latent Variable Model

In order to utilize multimodal -omics data, we built a joint model for the feature space. Figure 7b displays the stacking process by which our joint model was built. As a result of stacking, clustering multimodal data is not straightforward. Instead of comparing metrics between patients’ multimodal gene sets directly for clustering, a latent variable model was used to divide the data based on comparing samples to latent representations. Direct comparisons might employ other clustering approaches, like neighborhood clustering.

For latent variable modeling, we employed the expectation–maximization (EM) algorithm. The EM algorithm iteratively estimates the parameters of the latent variable model to maximize the expected complete data log-likelihood. The algorithm consists of two main steps: the expectation (E) step and the maximization (M) step.

During the E-step, the algorithm calculates the expected value of the latent variables given the observed data and current parameter estimates. Specifically, it computes the posterior probabilities of the latent variables, which, in this context, represent feature space distributions. Each data point is compared to each latent distribution and assigned a probability of belonging to each distribution.

In the M-step, the algorithm maximizes the expected complete data log-likelihood with respect to the model parameters, using the posterior probabilities computed in the E-step. This involves updating the parameters of the latent distributions to better fit the data assigned to them during the E-step. The process is repeated until convergence, typically when the increase in log-likelihood falls below a predefined threshold.

### 3.4. Data Clustering with Expectation–Maximization

The tool used for performing expectation–maximization is the DCEM algorithm, adapted in 2022 by Sharma et al. [39]. DCEM is available in the Comprehensive R Archive Network. DCEM implements an improved expectation–maximization routine (EM*) formulated by Kurban et al. [40] and the traditional expectation–maximization algorithm (EM) [41] for clustering. EM* expedites convergence via structure-based data segregation. EM* embeds the traditional EM into a nonlinear hierarchical data structure (heap) that allows the separation of data needing revisiting from data that do not. This enables the algorithm to focus on data that are more difficult to cluster, improving overall convergence speed and accuracy.

In EM*, during the E-step, each piece of data is compared to each latent distribution, and posterior probabilities are calculated as in traditional EM. However, EM* employs a heap structure to prioritize data points based on their likelihoods. Data ranked higher in the heap have a higher likelihood of belonging to the given Gaussian distribution, whereas data positioned lower are less likely to belong. This prioritization allows the algorithm to efficiently focus computational resources on the most challenging data points.

During the M-step, each Gaussian distribution is updated by maximizing the complete data log-likelihood, similar to the traditional EM approach. The heap associated with each latent distribution determines which data points are carried forward as active for the next iteration. This data-centric approach mitigates computational expense by ignoring less relevant data and activating more informative data. The values in the heap are informed by the likelihoods used for data and latent distributions, facilitating a more targeted optimization process.

The implementation of DCEM supports both random and K-means++ [42]-based initialization methods. The hierarchical structures within the routine differentiate high expressive data from low expressive data, where high expressiveness significantly impacts the optimization objective function.

With the implementation of both traditional and enhanced EM solvers, the DCEM package allows seamless testing and comparison of EM* to standard latent variable models that employ traditional EM solvers. This comparison can be easily made by realigning preprocessing steps. For instance, in Section 2, when comparing the runtime with iCluster (DCEM’s most similar latent variable method), only the runtimes of the EM routines are considered. The overall workflow for subtyping is summarized in Algorithm 1.

Finally, our subtyping was verified via survival analysis and the Kaplan–Meier test [43]. Further verification was based on a comparison to other subtyping methods and downstream subtype expression analysis. The Kaplan–Meier test (or Kaplan–Meier survival curve, Kaplan–Meier estimator) is a statistical method used primarily in cancer research to estimate the probability of survival over time for subtypes of disease patients or other study sub-populations. The Kaplan–Meier test calculates the probability of survival at different times by considering the number of patients at risk at each time point and the number of events occurring at that time. The estimator computes the probability of survival at each point, typically depicted as a step-like survival curve. The Kaplan–Meier test is a valuable tool in cancer research for understanding the survival outcomes of different cancer subtypes or treatment groups. In this subtyping context, the Kaplan–Meier test provides a metric for the validity of the proposed patient subtyping.

The log-rank test statistically compares the survival distributions of two or more groups. It evaluates whether there is a significant difference in the survival experience between these groups over time. The test works by comparing the observed and expected number of events at various time points, providing a *p*-value as a measure of statistical significance. A low *p*-value from the log-rank test indicates that the survival distributions of the groups are significantly different, supporting the distinctiveness of the identified subtypes and reinforcing the robustness of the subtyping methodology.
**Algorithm 1:** Multiomics data integration with DCEM.% load -omics data mRNA←load(TCGAmRNAdata) DNA←load(TCGADNAdata) miRNA←load(TCGAmiRNAdata) % feature selection as in Figure 7a mRNAfs←FSbyCox(mRNA) DNAfs←FSbyCox(DNA) miRNAfs←FSbyCox(miRNA) % initialize latent variable model as in Figure 7b multiomics←stack(mRNAfs,DNAfs,miRNAfs) % optimize latent distributions subtype_assignments←dcem(multiomics) **return **subtype_assignments

### 3.5. Subtype Expression Analysis

The first step of the subtype expression analysis was to identify the top differentially expressed genes of each subtype. An extension of limma in the CancerSubtypes Bioconducter package was used to perform this analysis [38]. The top 50 differentially expressed genes per subtype for the two cancers were identified for further downstream pathway analysis. Differentially expressed genes are calculated by establishing the log fold change in gene expression between two datasets, which, in this case, are healthy and tumor-affected patients. For this study, a log fold change of plus or minus 3.5 with *p*-value < 0.05 was considered a differentially expressed gene and was utilized for further downstream analysis.

Several R packages Several R packages were used to perform the functional enrichment analysis for each subtype. All softwares were run using R version 4.4.0. To conduct the GO and DISGINET pathway enrichment, the online tool DAVID was used [36], version v2024q4. In this study, the AmiGO database (version v2.4.26) was leveraged to identify overexpressed GO terms in each subtype for breast cancer [44]. The differentially expressed gene sets identified by the CancerSubtypes package (version 1.10.0) from TCGA were used as the basis for pathway enrichment [38].

To calculate functional similarity, GO Semantic Similarity was used. GO Semantic Similarity is an information content (IC)-based method that scales functional similarity from 0 to 1, with 0 being the least functionally similar and 1 being the most functionally similar [37] (version 2.32.0). The information is calculated by leveraging the AmiGO database of biological processes, molecular functions, and cellular components. Each of these categories contains entities known as GO terms, which comprise identifiers beginning with GO, and genes associated with each GO [44]. GO Semantic Similarity is calculated by measuring the number of overlapping genes between two GO terms and is one of the most widely used metrics to measure functional similarity between GO terms and gene products [37].

**Figure 8:**
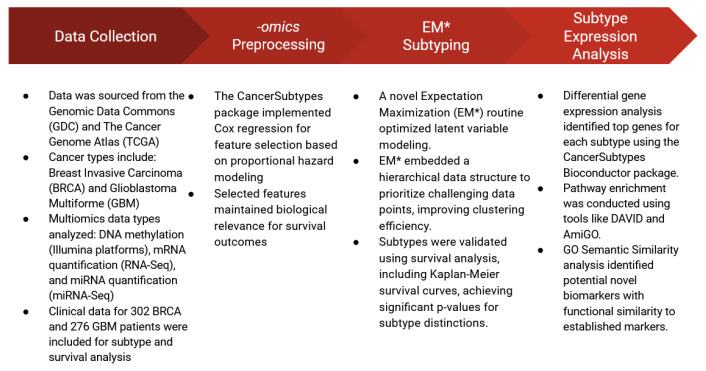
A summary of the multi-step workflow employed in this study, highlighting the data collected from TCGA, the process by which features were selected, the implementation of EM* for multiomics data integration, and the methods employed for subtype analysis.

Figure 8 illustrates the multi-step workflow employed in this study, including data collection, preprocessing of multiomics datasets, implementation of the improved EM* algorithm for clustering, and subsequent subtype expression analysis. This workflow emphasizes the integration of biologically informed feature selection with robust clustering methodologies to identify clinically relevant cancer subtypes.

## 4. Discussion

The development and application of the improved expectation–maximization (EM*) algorithm within the DCEM framework has demonstrated significant advancements in the field of multiomics data integration, particularly for cancer subtyping. The robustness of the EM* algorithm to incomplete patient data and its ability to enhance computational efficiency are key benefits that make it a promising tool for large-scale genomic studies.

The EM* algorithm, by leveraging a data-centric approach and heap structures, optimizes the computational resources required for clustering multiomics data. This is especially crucial given the exponential growth in the volume of multiomics data. The comparison of runtime expense between the EM* algorithm and the traditional EM routine used by iCluster underscores the potential of EM* in reducing computational costs. This is a significant achievement, as it allows for the scalability of the algorithm to larger datasets, making it feasible to handle extensive multiomics data without compromising performance.

The improved expectation–maximization routine has been shown to enhance the accuracy of cancer subtyping. This was evident from the survival analysis results, where EM* achieved lower *p*-values compared to traditional EM, indicating better-defined patient subtypes with distinct survival outcomes. The ability to achieve robust subtyping even with missing patient data further highlights the algorithm’s practical applicability in real-world scenarios where incomplete datasets are common.

The four groups informing the subtype analyses are the DCEM with EM* routine found in Figure 3b. The subtype expression analysis provided key biological insights into the functional differences between the identified cancer subtypes. Importantly, the upregulation of COL10A1 has been demonstrated to promote breast cancer progression and lead to poor prognosis in patients [45,46]. The downregulation of CDG300LG has been linked to brain metastatic cancer stemming from breast cancer patients [47]. The differential expression analysis of each subtype was consistent with current patterns of breast cancer progression. While every subtype has differential expression of COL10A1 and CDG300LG, each subtype has some unique genes that are differentially expressed but still have some significance to breast cancer.

In Subtype 1, the gene RGMA is downregulated, with a log2fold change of approximately −8. RGMA is known to be a tumor suppressor gene, not specific to breast cancer. A study by Li et al. demonstrated that the inhibition of RGMA leads to increased breast cancer tumor cell proliferation [48]. In fact, overexpression of RGMA has been shown to decrease breast cancer in humans [48]. Another gene downregulated within Subtype 1, KRT19, has been significantly linked to poor prognosis in breast cancer patients. A low expression of KRT19 in breast cancer patients was attributed to higher tumor cell proliferation [49]. In Subtype 2, we noted the upregulation of FMO2, which has been identified to be a marker of Cancer-Associated Fibroblast cells (CAFs) in breast cancer. FMO2 was found in other studies to be significantly upregulated in CAF populations [50]. We also noted the downregulation of CCL11 in Subtype 2, which is a well-documented immune cell proliferator, particularly in breast cancer tissue [51].

Subtype 3 had the upregulation of MMP13, a well-studied tumor marker in breast cancer cells. The upregulation of MMP13 has been linked to a poor prognosis in breast cancer patients [52]. In Subtype 4, we identified the upregulation of the HJURP gene, which is a marker for triple-negative breast cancer. A recent study demonstrated that the expression of HJURP is higher in triple-negative breast cancer than in other breast cancer subtypes. We also noted the upregulation of CASQ2, the overexpression of which was recently associated with a more aggressive form of breast cancer [53]. From this analysis, we see that, while each subtype identifies unique up- and downregulated genes, many of them have strong associations with breast cancer, further confirming the biomedical relevance of each subtype.

The relationship between genes in each subtype was quantified through GO Semantic Similarity analysis. For example, COL10A1 is a well-established biomarker for breast cancer [45]. The upregulation of this gene is present in three out of the four subtypes. Genes with a high functional similarity to COL10A1 may be further investigated for a linkage to breast cancer. In BRCA Subtype 1, we see that there is a strong GO Semantic Similarity relationship of 1 between COL10A1 and MATN2. The relationship between MATN2 and breast cancer is currently unclear; MATN2 is a protein-coding gene that is speculated to be involved in the formation of filamentous networks in the extracellular matrices of several tissues [54]. Other strong GO Semantic Similarity relationships can confirm well-studied biomarkers currently proven in the literature. A potential further work would be to investigate the relationship between MATN2 and breast cancer, since it has been revealed to have a strong functional relationship with a well-established breast cancer biomarker.

## 5. Conclusions

The implementation of the EM* algorithm within the DCEM framework represents an improvement in the field of multiomics data integration for cancer subtyping. The EM* algorithm significantly reduces computational costs associated with multiomics data integration, enabling the efficient handling of larger datasets. This makes it a viable option for large-scale genomic studies. The improved EM* routine links robust cancer subtypes with distinct survival outcomes, even in the presence of incomplete patient data. This robustness is critical for the practical application of the algorithm in clinical settings. The DCEM framework with the EM* algorithm facilitates the identification of key differentially expressed genes and enriched pathways, offering valuable insights into the biological underpinnings of cancer subtypes. These insights are crucial for developing personalized treatment strategies and advancing precision medicine. The identification of genes with high functional similarity to known biomarkers, such as MATN2 to COL10A1, opens new avenues for research into the roles of these genes in cancer progression. This can lead to the discovery of new biomarkers and therapeutic targets.

In conclusion, the DCEM framework with the EM* algorithm provides a powerful tool for multiomics data integration, offering enhanced computational efficiency, robust subtyping, and valuable biological insights. This study underscores the importance of continued advancements in optimization routines and data integration methods to fully harness the potential of multiomics data in cancer research and treatment.

Future research should focus on further validation of the EM* algorithm across different cancer types and multiomics datasets to generalize its applicability. Additionally, integrating the EM* algorithm with other advanced machine learning techniques, such as deep learning models, could further enhance its performance and utility. The exploration of the identified potential biomarkers and their roles in cancer progression also warrants in-depth biological studies to translate these findings into clinical applications.

## Figures and Tables

**Table 1:** Testing the robustness of survival analysis trials to vanishing patient data. Table values depict the average *p*-values across groups as a function of randomly removed patients for five trials.

Patients	Trial 1	Trial 2	Trial 3	Trial 4	Trial 5
276	3 × 10^−6^	3 × 10^−9^	2 × 10^−4^	2 × 10^−6^	8 × 10^−6^
265	2 × 10^−5^	9 × 10^−4^	1 × 10^−5^	6 × 10^−6^	1 × 10^−6^
255	4 × 10^−8^	2 × 10^−4^	5 × 10^−4^	7 × 10^−6^	6 × 10^−5^
245	5 × 10^−4^	2 × 10^−5^	2 × 10^−4^	1 × 10^−6^	0.001
235	4 × 10^−5^	4 × 10^−4^	9 × 10^−6^	3 × 10^−4^	0.05
225	1 × 10^−5^	3 × 10^−9^	4 × 10^−6^	1 × 10^−4^	1 × 10^−5^
215	8 × 10^−5^	4 × 10^−6^	1 × 10^−4^	0.003	2 × 10^−5^
205	4 × 10^−6^	8 × 10^−4^	5 × 10^−4^	0.002	0.001
200	1 × 10^−5^	3 × 10^−5^	4 × 10^−4^	0.004	3 × 10^−6^
150	9 × 10^−4^	3 × 10^−4^	5 × 10^−7^	0.003	0.009
100	0.04	0.003	0.5	0.004	2 × 10^−5^

## Data Availability

Data are contained within the article.
